# SARS-CoV-2 surveillance and testing: results of a survey from the Network of University Hospitals (NUM), B-FAST

**DOI:** 10.3205/dgkh000402

**Published:** 2021-11-22

**Authors:** Stephanie Heinemann, Anna Bludau, Hani Kaba, Percy Knolle, Hajo Grundmann, Simone Scheithauer

**Affiliations:** 1 Institute of General Medicine, University Medical Center Göttingen, Germany; 2 Local Task Force Network University Medicine (NUM), University Medical Center Göttingen, Germany; 3 Institute for Infection Control and Infectious Diseases, University Medical Center Göttingen, Germany; 4 Institute of Molecular Immunology and Experimental Oncology Klinikum rechts der Isar of the Technical University of Munich, Germany; 5 Institute for Infection Prevention and Hospital Hygiene University Medical Center Freiburg, Germany

**Keywords:** hospital, SARS-CoV-2, screening, surveillance, university hospital

## Abstract

**Background:** The B-FAST project of the National University Network (NUM) examines and records applied surveillance strategies implemented in hospitals i.a., to protect patients and employees from SARS-CoV-2 infection.

**Methods:** Infection control physicians in German university hospitals (UK), as well as non-university hospitals (NUK; Bavaria, Lower Saxony) were surveyed in March 2021 regarding SARS-CoV-2 testing/surveillance strategies in a cross-sectional study using a standardized online questionnaire. The focus was on screening strategies taking into account the “test” methods used (case history, PCR, antigen, antibody test).

**Results:** The response rate was 91.7% (33/36) in UK and 11.3%–32.2% in NUK. Almost all hospitals (95.0%) performed a symptom and exposure check and/or testing upon inpatient admission. Non-cause-related testing (screening) of health care workers in COVID wards was preferably done by PCR in UK (69.7% PCR; 12.1% antigen), while NUK (29.9% PCR; 49.3% antigen) used antigen testing more frequently. Regardless of the type of facility, about half of the respondents rated the benefit of screening higher than the effort (patients: 49%; employees: 45%).

**Conclusion:** Testing/surveillance strategies find a high level of acceptance at German hospitals and are generally carried out in accordance with the national testing strategy with differences depending on the level of care.

## Zusammenfassung

**Hintergrund:** Im „Bundesweiten Forschungsnetzwerk zur Angewandten Surveillance und Testung“ (B-FAST) werden anwendungsbezogene Strategien geprüft und erfasst, die u.a. in Krankenhäusern zum Schutz von Patient*innen und Beschäftigten vor SARS-CoV-2 implementiert wurden.

**Methoden:** Im März 2021 wurden in einer Querschnittstudie mit einem standardisierten Online-Fragebogen die leitenden (Krankenhaus-)Hygieniker*innen in deutschen Universitätskliniken (UK), sowie Nicht-Universitätskliniken (NUK) in Bayern und Niedersachsen zu SARS-CoV-2 Test- und Surveillance-Strategien befragt. Im Fokus standen Screening-Strategien unter Berücksichtigung der eingesetzten „Test“-Methoden (Anamnese, PCR-, Antigen-, Antikörpertest).

**Ergebnisse:** Der Rücklauf liegt bei 91,7% (33 von 36) aus den UK, bei 32,2% (37 von 115) aus den niedersächsischen NUK und bei 11,3% (30 von 265) aus den bayrischen NUK. Nahezu alle Kliniken (95,0%) führen bei der Aufnahme stationärer Patient*innen einen Symptom/Expositionscheck und/oder eine Testung durch. Das nicht-anlassbezogene Testen (Screening) von Beschäftigten im COVID-Bereich erfolgte in UK (69,7% PCR; 12,1% Antigen) bevorzugt durch PCR, während NUK (29,9% PCR; 49,3% Antigen) häufiger Antigentests einsetzten. Unabhängig von der Einrichtungsart bewerten etwa die Hälfte der Befragten den Nutzen von Screening höher als den Aufwand (bei Patient*innen 49,0%; bei Beschäftigten 45,0%).

**Fazit:** Test- und Surveillance-Strategien finden an deutschen Kliniken hohe Akzeptanz und werden grundsätzlich in Anlehnung an die nationale Teststrategie – mit Unterschieden je nach Versorgungsstufe – durchgeführt.

## Key points

- Non-cause-related screening as well as symptom and exposure checks are carried out on inpatients regardless of the level of care.
- Screening of outpatients is performed in 78.0% of clinics with significant differences between levels of care.
- Non-cause-related screening is administered to employees in three different situations:

1.
  1.
    1. in the COVID-19 setting,
  2.
    1. regardless of the setting, and
  3.
    1. when requested by employees, with considerable heterogeneity across facility types.

- The method of choice in university hospitals is PCR, in non-university hospitals rather antigen detection.
- Regardless of the level of care, about half of the respondents assessed that the benefits of screening measures exceed the costs. A risk-benefit and cost-benefit assessment of different strategies would be desirable – also to develop recommendations as best practice beyond the COVID-19 pandemic.

## Background

During the COVID-19 pandemic, German hospitals implemented new strategies in the field of infection prevention to protect patients and employees in as short a time as possible. Within the “Federal Research Network for Applied Surveillance and Testing” (B-FAST), these are recorded and used as a basis for action recommendations. Such strategies aim to contain the spread of a certain pandemic pathogen through hygiene and public health measures. 

A hospital’s central measure for containing the spread of pathogens is its testing and surveillance strategy. The early identification of SARS-CoV-2–infected persons without typical COVID-19 symptoms is a particular challenge. Their timely isolation can be an important factor in preventing further spread of infection among patients, workers, and relatives [1], [2].

The German “Nationale Teststrategie” (national testing strategy) describes testing as “[...] the basis for timely detection and treatment of infections, for breaking chains of infection, and for protecting the health care system from becoming overburdened” [3]. It also specifies who should be tested for a SARS-CoV-2 infection. In general, hospitals had already established and adapted their own procedures before the publication of the first “Corona-Testverordnung” (COVID-specific testing regulation) on June 8, 2020 [4].

Differences in the implementation of these regulations could be due to specific local material, human and infrastructural resources, and regional pandemic events, among other things. In 2020, for example, the 7-day incidence in Bavaria was on average higher than in Lower Saxony [5]. Based on this difference, university hospitals in Germany, as well as non-university hospitals in Lower Saxony and Bavaria, were selected for a survey to obtain an overview with as much contrast as possible. Among other things, the survey focused on the non-cause-related screening strategies, taking into account the “test” methods used (history taking, PCR, antigen, antibody tests).

The following questions were addressed:

1. Which cause-related and non-cause-related screening and testing strategies are pursued for patients and employees at German hospitals?
2. Which SARS-CoV-2 tests (PCR, antigen, antibody) are carried out on patients and employees?
3. What questions about COVID symptoms and exposition are patients asked when they enter the hospital building?
4. How do the respondents assess the relationship between effort and benefit of non-cause-related screening tests?

The results were evaluated for potential differences between university hospitals and non-university hospitals. An assessment of the respondents on their chosen testing strategy was part of the survey. The results are particularly relevant against the current backdrop of the updated “Landes Corona Verordnung” (state specific regulations), which includes mandatory rules (“3G”; “2G”).

## Methods

### Study design and participants

In a cross-sectional study with a standardized questionnaire, the heads of (hospital) hygiene departments in German hospitals were asked about their SARS-CoV-2 testing and surveillance strategies. 

The sample consisted of three groups: 1) total number of university hospitals (UK; n=36), 2) non-university hospitals (NUK) in Lower Saxony (n=115), and 3) NUK in Bavaria (n=265). The two states were chosen based on their different levels of being affected by the COVID-19 pandemic over the past year. The surveyed units were the organizations and not individuals within the organization. A detailed description of the questionnaire development, as well as strategies for reaching the target group, is given in the Appendix .

The questionnaire was designed and tested in an interdisciplinary process with representatives from the fields of microbiology, virology, hospital hygiene, and public health. The survey was conducted in March 2021 using the online survey tool *LimeSurvey*. 

The survey protocol was officially approved by the data protection commissioner on Febuary 8, 2021 (B-FAST/tl) and by the ethics committee of the University Medical Center Göttingen (UMG) on January 29, 2021 (5/2/25). 

### Statistical analysis

The data were analyzed in IBM SPSS Statistics 26 and stratified according to the level of care (UK and NUK). The survey data were analyzed descriptively within these two categories. The results are presented in percentages. 

## Results

### Return

Of 416 questionnaires sent out, 100 were filled in completely, representing a response rate of 91.7% (33 out of 36) from UK, 32.2% (37 out of 115) from Lower Saxony NUK and 11.3% (30 out of 265) from Bavarian NUK. 

In the following, research questions 1 to 4 are addressed individually below, and the results are presented in relation to the entire sample by level of care (i.e., university hospitals vs. non-university hospitals).

### 1. Cause-related and non-cause-related screening and testing strategies

#### For patients

Non-cause-related testing (screening) is defined as testing of persons without symptoms or exposure. In the majority of hospitals (95.0%), non-cause-related testing takes place upon admission for inpatients and during the consultation (54.0%) for outpatients. Significant differences between the levels of care were evident (Table 1 (Tab. 1)). 

#### For employees

While all UK workers are tested by PCR for symptoms and exposure (Table 2 (Tab. 2)), only about half of NUK perform PCR testing in these constellations (52.2% in case of symptoms, 50.8% in case of exposure/contact). Antigen testing is used in case of symptoms and exposure/contact in 12.1% of UK and in about half of NUK (56.7% in case of symptoms, 53.7% in case of exposure). This suggests that UK rarely use the antigen test as a rapid screening tool and always confirm it by PCR. The NUK use the tests alternately.

Non-cause-related testing varies highly across facility types for employees in three different situations:

1. in the COVID-19 setting,
2. regardless of the setting, and
3. when requested by the employees.

True random sampling is used infrequently across hospitals (0.0%–12.1%).

In the COVID-19 setting, PCR screening is offered at approximately two-thirds of UK (69.7%) and one-third (29.9%) of NUK. Around half of the UK (48.5%) offer PCR testing to their employees on request. Non-cause-related PCR testing is generally less frequent in NUK (9.0–29.9%). 

Antigen tests are used more frequently in all three situations in NUK than UK. The most common situation for antigen testing in all hospitals is “regardless of the setting” (30.3% UK, 62.7% NUK). 

### 2. Use of SARS-CoV-2 diagnostics (PCR, antigen, antibody)

In general, testing of patients is most frequently conducted by PCR (92.0%), followed by antigen testing (78.0%), and only rarely by antibody testing (34.0%). In some cases, there are major differences in the use of these tests among the various groups of people, such as visitors, students, and permanent as well as marginally employed persons (e.g. so-called “mini-jobs” with a maximum gross monthly wage of Euro 450) (Table 3 (Tab. 3)). 

### 3. Questioning of the patients when entering the hospital building

In 95.0% of the hospitals, a routine survey of symptoms and exposures takes place for patients who want to enter the hospital building (Table 4 (Tab. 4)). There are differences between the levels of care, with more frequent questions about quarantine and isolation in UK. In NUK, on the other hand, exposition and body temperature are recorded more frequently.

### 4. Assessment of effort and benefits of screening strategies for patients and employees

**Figure 1:**
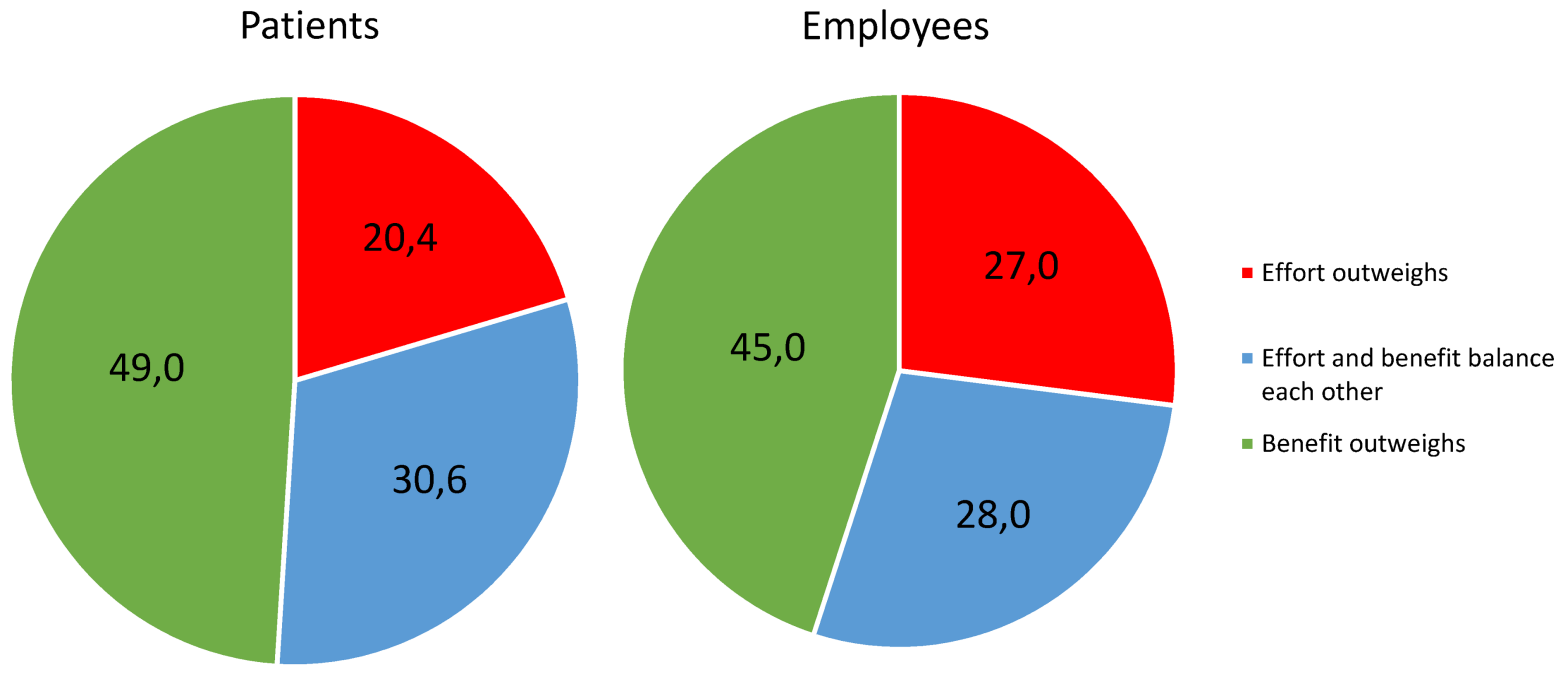
Ratio of effort to benefit for non-cause-related testing of patients and employees from the point of view of the participants (Expertise Infection control/prevention)

Almost all hospitals were able to identify at least one SARS-CoV-2 infected patient (92.0%) or employee (85.0%) through their screening strategies (data not shown). Regardless of the level of care, about half of the respondents rated that the benefits of screening exceeded the costs (49.0% for patients; 45.0% for employees) (Figure 1 (Fig. 1)). 

## Discussion

The testing and surveillance strategies in Germany were solidly established by March 2021. Concerning the design, there are differences between the levels of care. 

The aim of the National Testing Strategy is, among other things, to standardize the testing of asymptomatic persons, especially in health care facilities, and thus to identify infected persons at an early stage [3]. It guides the implementation of testing and surveillance activities. It is evident that the hospitals had either developed analogous strategies or adapted them to the National Testing Strategy. 

Especially for highly vulnerable patients or for patients who are at increased risk of infection, early detection of an infection is important so that measures can be initiated in time. Non-cause-related testing was performed more frequently in UK for these groups of patients, possibly because NUK tend to treat such patients less frequently. 

It appears essential that the non-occasion-related screening be carried out very extensively, irrespective of the level of care. Half of the respondents rated that the benefits of screening exceeded the costs, even considering the high logistical effort and material, as well as personnel costs behind the implementation of such screening strategies. Initial evaluations of effectiveness are already available [6], [7]. Holistic analyses, including health-economic considerations of the entire process chain, are needed so that a sound basis for regulations can be provided for the further course of the pandemic as well as future pandemics. 

It should also be taken into account that almost all hospitals, regardless of the level of care, conduct a questionnaire on symptoms and/or exposure of patients entering the hospital building. This “COVID history” could be a very effective and possibly also efficient tool for early detection of asymptomatic or pre-symptomatic infected patients and thus contribute to the prevention of transmission to other patients and employees. The efficiency of this tool is being investigated in initial studies within B-FAST [8]. 

Many hospitals offer non-cause-related employee testing as well. This is remarkable, since the legal obligation for companies to offer testing at least twice a week for all employees working in the hospital was installed after the survey had been conducted. Here, the UK preferentially offer PCR testing to a relevant extent beyond the recommendations. The question of refinancing options that arise here was not part of the survey.

Overall, the use of PCR testing in UK dominates, which can be interpreted as an expression of the increased availability of on-site diagnostics. Financial considerations may also have played a role. These factors should be considered with regard to National Testing Strategies in the context of generic Pandemic Preparedness strategies. The National Testing Strategy favors the use of PCR due to its higher test quality. The use of antigen testing should only be considered as a supplement. According to the National Testing Strategy, a positive or ambiguously negative antigen test should always be checked with a PCR test [3]. 

For cause-related testing (symptoms or contact), all UK and about half of the NUK use PCR testing. In the other half of NUK, antigen testing is used for these cases as well. There is little overlap here, suggesting that few hospitals use both testing methods for these cases. It is also possible that NUK, which cannot offer PCR testing on site, ask their employees to have PCR testing performed externally. The lower sensitivity of antigen tests appears clinically relevant here. We can assume a higher number of undetected SARS-CoV-2–infected patients and employees in NUK than in UK. Whether this is relevant for transmission chains and outbreaks cannot be proven based on this data. 

Due to the increasing vaccination rate within the population, the antigen test may lose quality, especially if vaccination leads to reduced replicative activity and if the formation of antigen-antibody complexes is relevant. Since this is apparently not the case with the currently circulating delta variant [9], the antigen test – with its known limitations – can continue to be used as a rapidly available, easy-to-perform diagnostic tool. Currently, there is no differentiated specification in the National Test Strategy regarding this development. A renewed survey on possible process changes in the test strategy of the hospitals would be interesting in the future. 

### Strengths and limitations of the study

In principal, the known limitations of studies based on the subjective assessment of experts about a current topic apply. In addition, the survey represents a snapshot. Methodologically, an attempt has been made to optimize the instrument in the context of pretests by experts outside the basic population. The excellent response rate of the UK and the adequate response rate of the NUK are strengths that should also be mentioned.

These survey results are part of an iterative survey strategy and feed into the development of both recommendations and further survey tools. They can be used to advise policy-makers and other stakeholders, and as a quick survey. The “expert evidence” obtained in this way is by no means intended to replace a systematic literature analysis, but merely to provide an additive assessment in times of rapidly changing requirements and to serve as a guide for action. 

## Conclusions and implications for practice

The results show a high implementation of infection control measures in German hospitals. There are differences in the strategies between UK and NUK as well as in the application among patients and employees. Questions regarding the refinancing of the personnel and material infrastructure remain unanswered. Further research should focus on evaluating the cost-benefit ratio of the strategies in order to develop best-practice measures beyond the COVID-19 pandemic. 

## Notes

### Competing interests

The authors declare that they have no competing interests.

### First authorship

Heinemann S. and Bludau A. share the first authorship.

### Acknowledgement

The authors would like to thank Dr. Matthias Pulz, Dr. Katja Hille and Dr. Martina Scharlach from the NLGA for their support in preparing and sending out the survey.

Thanks to Priv.-Doz. Dr. Reiner Schaumann, Dr. Ulrike Löderstadt from the Institute for Infection Control and Infectious Diseases at UMG, as well as Dr. Thomas Hauer, Dr. Christian Brandt and Prof. Dr. Markus Dettenkofer for evaluating the survey instrument. Last but not least, thanks to Dr. Thomas Langbein, UMG data protection officer, for advising on data protection law, and Prof. Dr. Jürgen Brockmöller, chairman of the UMG Ethics Committee, for ensuring an ethical approach. 

## Supplementary Material

Description of the development and mailing of the questionnaire

## Figures and Tables

**Table 1:**
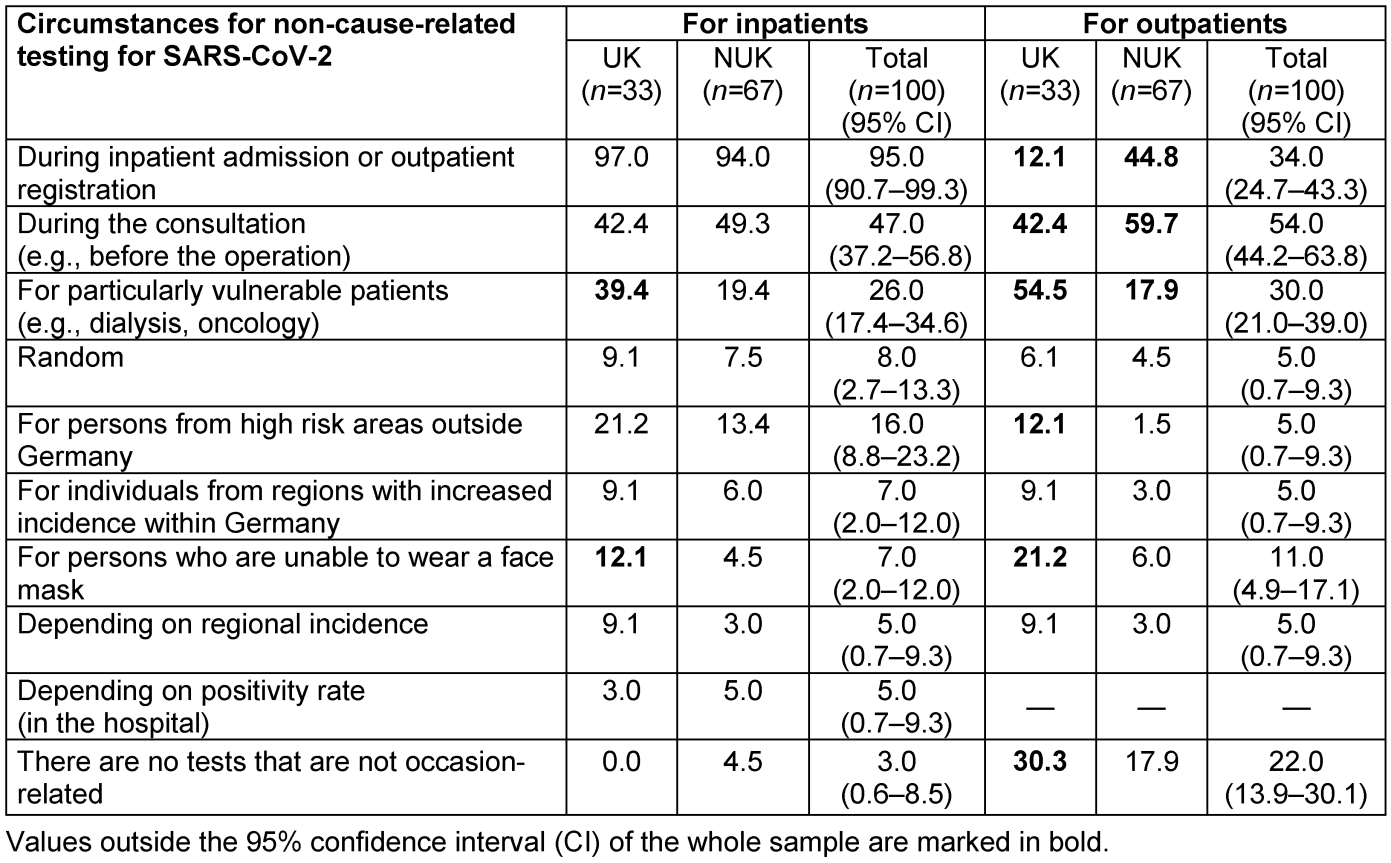
Circumstances for non-cause-related testing for SARS-CoV-2 infections in inpatients and outpatients stratified by level of care; all data in percent; multiple answers possible

**Table 2:**
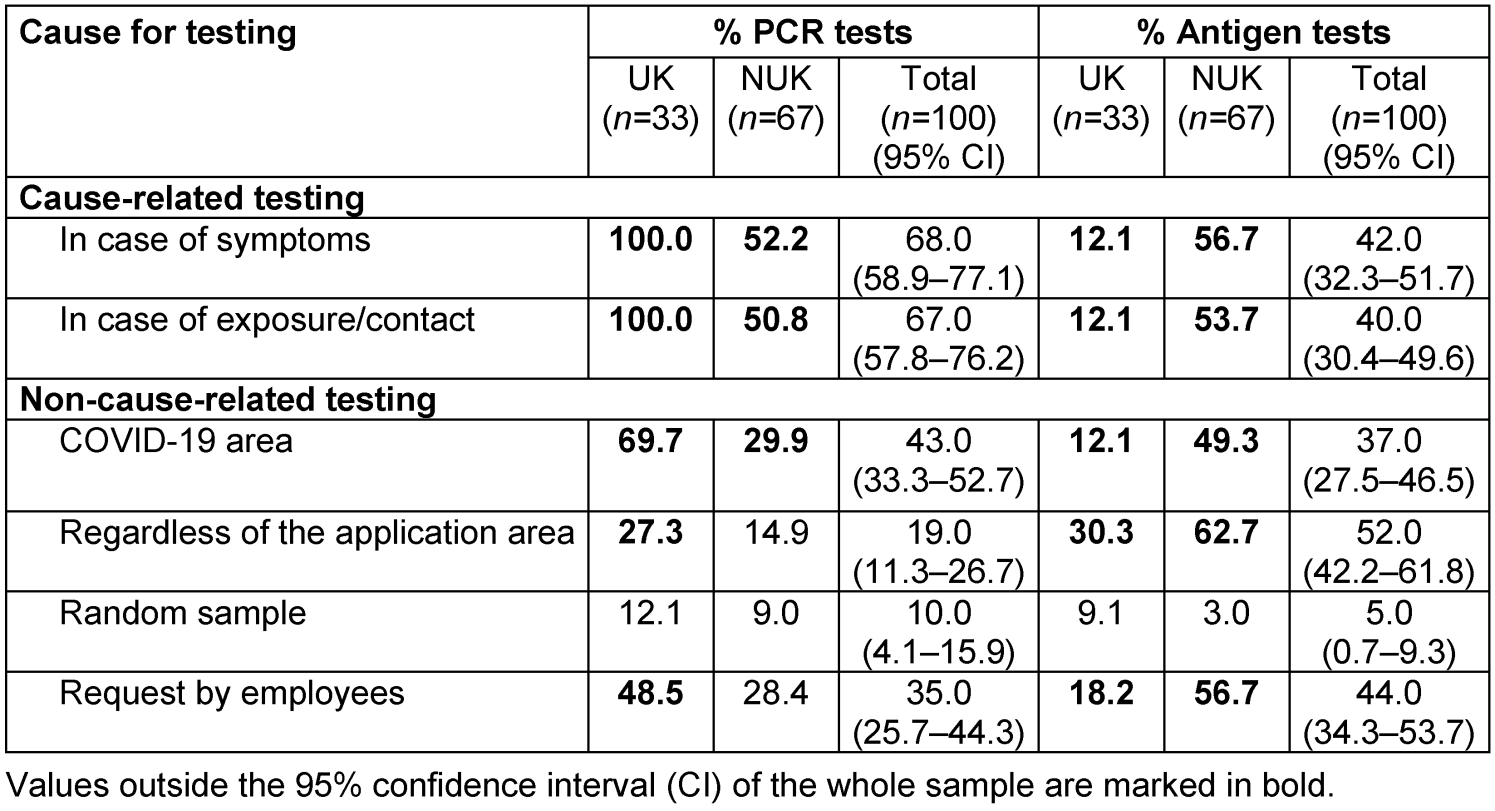
Strategy for specific cases of cause-related or non-cause-related testing for SARS-CoV-2 infections in employees stratified by level of care; all data in percent; multiple answers possible

**Table 3:**
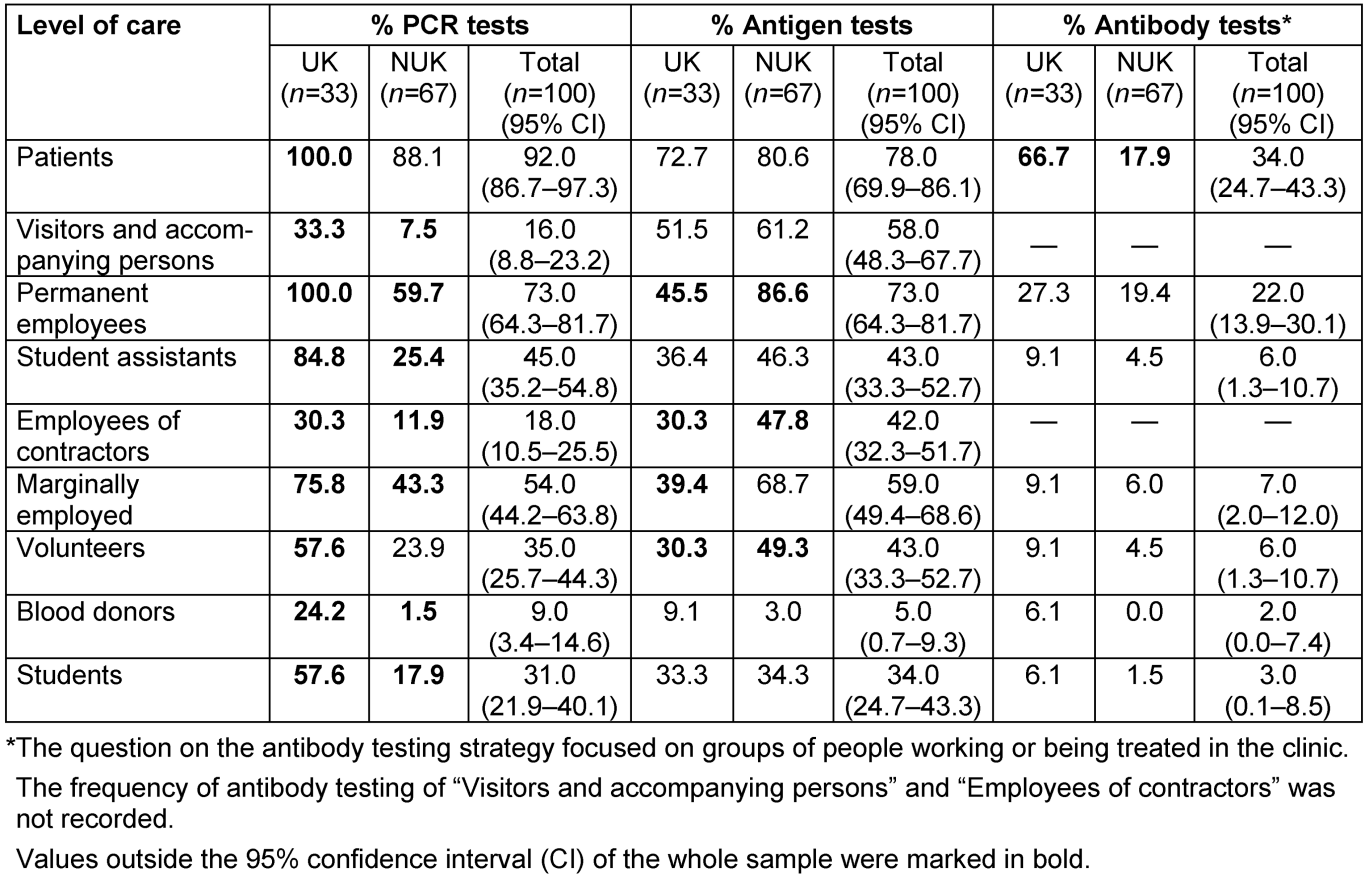
SARS-COV-2 testing services stratified by level of care; all data in percent; multiple answers possible

**Table 4:**
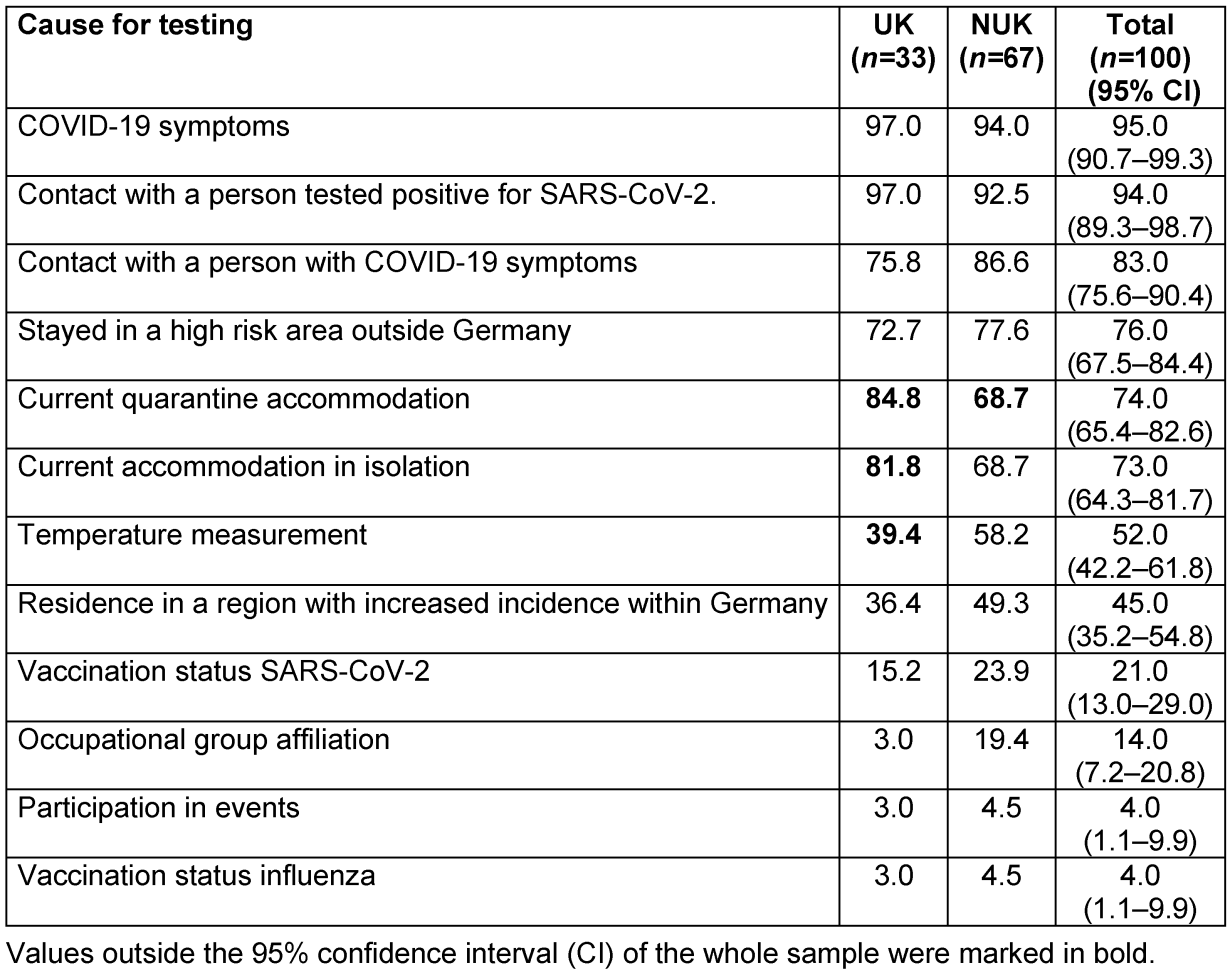
Survey of patients entering the hospital building stratified by level of care; all data in percent; multiple answers possible
