# Supplementary material for: SARS-CoV-2 surveillance and testing: results of a survey from the Network of University Hospitals (NUM), B-FAST
Source: GMS Hyg Infect Control. 2021 Nov 22;16:Doc31. doi: 10.3205/dgkh000402 (PMC8662743; doi:10.3205/dgkh000402)
Supplement: Description of the development and mailing of the questionnaire [file HIC-16-31-s-001.pdf]

Appendix

Description of the development and mailing of the questionnaire

The questionnaire was designed in an interdisciplinary process with representatives from the fields of microbiology, virology, infection control and public health. This was followed by a pretest with five experts outside the sample to ensure the comprehensibility, relevance and logic of the survey. After revising the questionnaire, the final version was incorporated into the online survey tool *LimeSurvey.* The functionality of the online questionnaire was tested in several internal runs.

The procedure for sending out the invitation letters differed between the UK and NUM. Hygiene managers in the UK were contacted through their respective NUM task forces and asked to participate in the survey. Prior to this, the task forces were informed in an online event about the background and objectives of the study and their role as facilitators. Hygiene representatives in the UK received an individual access key with the link to the survey. This key cannot be linked to the responses and was only used to track which UK had already participated in the survey. In this way, it was possible to remind people to participate. In addition, it also ensured that each UK participated only once. In the case of UK with several sites, the hygiene officer of the site with the highest number of beds would always take part in the survey on behalf of the other site.

The contact with NUK hygiene managers was established in cooperation with the Lower Saxony State Health Office (NLGA) and the Bavarian State Office for Health and Food Safety (LGL). The initial invitation letter on 01.03.2021 was followed by two reminders (10.03.2021 and 16.03.2021) to all clinics and an additional reminder (22.03.2021) to the UK. In order to inform the participants comprehensively and in an understandable way about the study, an information video was created and a link to it in the invitation and reminder letters was given.

Available from: Heinemann S, Bludau A, Kaba H, Knolle P, Grundmann H, Scheithauer S. SARS-CoV-2 surveillance and testing: results of a survey from the Network of University Hospitals (NUM), B-FAST. GMS Hyg Infect Control. 2021;16:Doc31. DOI: 10.3205/dgkh000402
